# 

**Published:** 1872-07

**Authors:** 


### Books and Pamphlets Received.

Lectures on the Principles and practice of Physic. Delivered at King's College, London. By Sir Thomas Watson, Bart. M. D., F. R. S. In two Volumes. From the fifth English Edition. Edited with additions, by Henry Hartshorne A. M., M. D. Philadelphia: Henry C. Lea 1872. Buffalo: T. Butler & Son.

A Year-Book of Therapeutics, Pharmacy and Allied Sciences. Edited by Horatio C. Wood, Jr., M. D. New York: Wm. Wood & Co. 1872. Buffalo: Breed, Lent & Co.

Lectures on Food. By H. Letheby, M. B., M. A., Ph. D., etc. New York: Wm. Wood & Co. 1872. Buffalo: Breed, Lent & Co.

Archives of Opthalmology and Otology. Edited by Prof. H. Knapp, M. D. New York, and Prof. S. Moos, M. D., Heidelberg. New York: Wm. Wood & Co. 1872.

Thirty-Fourth Annual Report of the Superintendant of Education of the City of Buffalo. For the year 1871.

Annual address delivered before the Medical Association of Central New York, June 18th, 1872. By the President B. L. Hovey, M. D., of Rochester, and an Essay on Asiatic Cholera. By W. S. Ely, M. D., of Rochester. Rochester: Erastus Darrow, 1872.

A Plea for the Antiphlogistic Treatment of Disease. By Edward Montgomery, M. D. Reprint from St. Louis Medical Archives, July 1872.

